# Molecular Characterization of Pneumococcal Isolates from Pets and Laboratory Animals

**DOI:** 10.1371/journal.pone.0008286

**Published:** 2009-12-14

**Authors:** Mark van der Linden, Adnan Al-Lahham, Werner Nicklas, Ralf René Reinert

**Affiliations:** 1 Department of Medical Microbiology, National Reference Center for Streptococci, University Hospital (RWTH), Aachen, Germany; 2 School of Applied Medical Sciences, German-Jordanian University, Amman, Jordan; 3 German Cancer Research Centre, Heidelberg, Germany; Charité-Universitätsmedizin Berlin, Germany

## Abstract

**Background:**

Between 1986 and 2008 *Streptococcus pneumoniae* was isolated from 41 pets/zoo animals (guinea pigs (n = 17), cats (n = 12), horses (n = 4), dogs (n = 3), dolphins (n = 2), rat (n = 2), gorilla (n = 1)) treated in medical veterinary laboratories and zoos, and 44 laboratory animals (mastomys (multimammate mice; n = 32), mice (n = 6), rats (n = 4), guinea pigs (n = 2)) during routine health monitoring in an animal facility. *S. pneumoniae* was isolated from nose, lung and respiratory tract, eye, ear and other sites.

**Methodology/Principal Findings:**

Carriage of the same isolate of *S. pneumoniae* over a period of up to 22 weeks was shown for four mastomys. Forty-one animals showed disease symptoms. Pneumococcal isolates were characterized by optochin sensitivity, bile solubility, DNA hybridization, pneumolysin PCR, serotyping and multilocus sequence typing. Eighteen of the 32 mastomys isolates (56%) were optochin resistant, all other isolates were optochin susceptible. All mastomys isolates were serotype 14, all guinea pig isolates serotype 19F, all horse isolates serotype 3. Rats had serotypes 14 or 19A, mice 33A or 33F. Dolphins had serotype 23F, the gorilla serotype 14. Cats and dogs had many different serotypes. Four isolates were resistant to macrolides, three isolates also to clindamycin and tetracyclin. Mastomys isolates were sequence type (ST) 15 (serotype 14), an ST/serotype combination commonly found in human isolates. Cats, dogs, pet rats, gorilla and dolphins showed various human ST/serotype combinations. Lab rats and lab mice showed single locus variants (SLV) of human STs, in human ST/serotype combinations. All guinea pig isolates showed the same completely new combination of known alleles. The horse isolates showed an unknown allele combination and three new alleles.

**Conclusions/Significance:**

The isolates found in mastomys, mice, rats, cats, dogs, gorilla and dolphins are most likely identical to human pneumococcal isolates. Isolates from guinea pigs and horses appear to be specialized clones for these animals. Our data redraw attention to the fact that pneumococci are not strictly human pathogens. Pet animals that live in close contact to humans, especially children, can be infected by human isolates and also carriage of even resistant isolates is a realistic possibility.

## Introduction

*Streptococcus pneumoniae* is the major causative pathogen of community-acquired respiratory tract infections (RTIs) in humans, including community-acquired pneumonia, acute otitis media and acute maxillary sinusitis. It is also the cause of severe invasive infections like meningitis and bacteremia in both children and adults. Most humans, especially young children (0–5 years of age), are likely to be colonized at least once during their lifetime with pneumococci that are spread by droplet infection [1]. Pneumococcal-related disease is usually preceded by colonization of the nasopharynx. Therefore, pneumococcal carriage is believed to play an important role in horizontal spread of *S. pneumoniae* in the community.

In general, *S. pneumoniae* is considered to be a human pathogen only, despite there being established mouse and rat models for various *S. pneumoniae-*caused diseases [2]. For example, BALB/c mice are used as models for pneumococcal pneumonia and meningitis [3], and rats for pneumococcal otitis media [4]. Transmission of other streptococcal species from animals to humans is well-documented, in particular for *S. equi* subsp. *zooepidemicus*, *S. canis*, *S. suis*, *S. porcinus* and *S. phocae* [5], [6], [7], [8], [9]. Pneumococci are naturally competent organisms and can easily exchange DNA in their natural habitat, the human mouth and throat. This habitat is populated by various streptococcal species, which form a ‘gene pool’ out of which the pneumococci can recruit their resistance genes. Gene transfer and mosaic genes have been intensively reported for *S. pneumoniae, S. oralis, S. sanguis* and *S. mitis* [10].

Multilocus sequence typing (MLST) produces unambiguous molecular typing data [11], [12]. The MLST technique is highly portable as any laboratory can compare the sequences of the seven loci in their isolates with those in a central database (www.mlst.net) and thereby obtain the allelic profile of each isolate. MLST results in data giving insight into the clonal relatedness of bacterial isolates.

In the present study, we characterized a large collection of *S. pneumoniae* from pets and laboratory animals including mastomys, guinea pigs, cats, mice, rats, horses, dogs, dolphins and a gorilla. Our findings show that *S. pneumoniae* is capable of colonizing and infecting animal hosts and raises the question whether these animals, which as pets are often in close contact to children, serve as an extra-human reservoir for *S. pneumoniae*.

## Results

During routine checks of animals in the animal facilities of the German Cancer Research Center (DKFZ) in Heidelberg, Germany, α-hemolysing streptococci were isolated from multimammate mice (*Mastomys coucha*, n = 32), rats (*Rattus norwegicus* F344, n = 4), mice (*Mus musculus* C57BL/6, n = 6) and guinea pigs (*Cavia porcellus*, n = 2). Isolates from 41 pets comprising guinea pigs (n = 17), cats (*Felis domesticus*, n = 12), dogs (*Canis lupus familiaris*, n = 3), horses (*Equus caballus*, n = 4) and rats (*Rattus rattus*, n = 2) were received from a veterinary medical laboratory (Vet Med Labor GmbH) in Ludwigsburg, Germany and the Department of Infectious Diseases and Immunology (VMDC) of the Faculty of Veterinary Medicine of the University of Utrecht, the Netherlands. Two isolates from dolphins (*Tursiops truncatus*) were obtained from a zoo in Nuremberg, Germany and one isolate from a gorilla (*Gorilla gorilla*) was from a zoo in Kerkrade, the Netherlands.

Two mastomys suffered from conjuntivitis. Two guinea pigs (laboratory animals) were C4-immune deficient, and one of them suffered from peritonitis and sepsis and died. All (n = 17) pet guinea pigs were obviously ill: RTI (n = 10), otitis media (n = 1), abcesses (n = 2), undefined illness (n = 4). Both pet rats were ill (one with RTI, one with unknown disease). Seven pet cats and three pet dogs suffered from RTI. The five other pet cats suffered from otitis media, otitis externa, conjunctivitis, polyarthritis and meningitis respectively. The latter animal died. Three horses had RTI, for one there were no detailed diagnostic data available. The two dolphins were zoo animals that died without showing clear symptoms of disease, but autopsy showed pneumococci in lung biopsies. The gorilla suffered from acute infection with inflammated tonsilli with purulent excretion.

Mastomys isolates were obtained from swabs taken from nose (n = 14), eye (n = 10), nose and eye (n = 2), lung (n = 1) and trachea (n = 1). Carriage isolates were obtained from eye swabs. Guinea pig isolates were from swabs from nose (n = 10), ear (n = 2) and wound, abcess, lung, trachea, abdomen, bulla tympanica (one each). For one animal the origin of the material could not be traced anymore. Isolates from laboratory mice an rats were all from nose swabs. For the two pet rats isolates wereobtained from lung and wound swabs (one animal each). For the cats islates were obtained from swabs from nose (n = 5), thorax (n = 2), and ear, wound, throat, synovia (one animal each). For the cat that suffered from meningitis and died the isolate was obtained from a brain biopsy. The three dog isolates were obtained from nose swabs. Horse isolates were obtained from nose swabs (n = 3) and BAL (n = 1). The two isolates from dolphins were obtained from lung-tissue biopsies. The gorilla isolate was obtained from purulent secretion material (**Table 1**).

**Table 1:** Characteristics of 85 *Streptococcus pneumoniae* isolates from pets and laboratory animals.

Isolate number	Animal	Year of isolation	country	origin	Diagnosis	Site of isolation	Opto Test	Serotype	MLST
24073	mastomys 1	2003	Germany	DKFZ	no clinical symptoms	nose	R	14	15
24074	mastomys 2	2003	Germany	DKFZ	no clinical symptoms	nose	S	14	15
24080	mastomys 3	2003	Germany	DKFZ	no clinical symptoms	nose	S	14	15
22877	mastomys 4	2003	Germany	DKFZ	no clinical symptoms	nose	R	14	15
22878	mastomys 5	2003	Germany	DKFZ	no clinical symptoms	nose	R	14	15
22879	mastomys 6	2003	Germany	DKFZ	no clinical symptoms	trachea	R	14	15
22875	mastomys 7	2004	Germany	DKFZ	no clinical symptoms	nose	R	14	15
22876	mastomys 8	2004	Germany	DKFZ	no clinical symptoms	nose	R	14	15
22881	mastomys 9	2004	Germany	DKFZ	no clinical symptoms	nose	R	14	15
22882	mastomys 10	2004	Germany	DKFZ	no clinical symptoms	lung	S	14	15
22883	mastomys 11	2004	Germany	DKFZ	no clinical symptoms	nose	R	14	15
22884	mastomys 12	2004	Germany	DKFZ	no clinical symptoms	nose	R	14	15
27110	mastomys 13	2005	Germany	DKFZ	no clinical symptoms	nose	S	14	15
22955	mastomys 14	2005	Germany	DKFZ	no clinical symptoms	nose	R	14	15
22956	mastomys 15	2005	Germany	DKFZ	no clinical symptoms	nose	R	14	15
26267	mastomys 16	2005	Germany	DKFZ	no clinical symptoms	eye	R	14	15
27072	mastomys 17	2006	Germany	DKFZ	no clinical symptoms	nose/eye	S	14	15
27673	mastomys 18	2006	Germany	DKFZ	no clinical symptoms	eye	R	14	15
27857	mastomys 19	2006	Germany	DKFZ	no clinical symptoms	eye	R	14	15
28293	mastomys 20	2006	Germany	DKFZ	no clinical symptoms	nose/eye	R	14	15
28417	mastomys 21	2006	Germany	DKFZ	no clinical symptoms	eye	R	14	15
28418	mastomys 22	2006	Germany	DKFZ	no clinical symptoms	eye	S	14	15
28419	mastomys 23	2006	Germany	DKFZ	no clinical symptoms	eye	R	14	15
28420	mastomys 24	2006	Germany	DKFZ	no clinical symptoms	eye	S	14	15
28421	mastomys 25	2006	Germany	DKFZ	no clinical symptoms	eye	S	14	15
28422	mastomys 26	2006	Germany	DKFZ	no clinical symptoms	eye	S	14	15
29791	mastomys 27	2006	Germany	DKFZ	conjunctivitis	eye	S	14	15
33432	mastomys 28	2008	Germany	DKFZ	conjunctivitis	nose	R	14	15
27703	mouse (C57BL/6) 1	2006	Germany	DKFZ	no clinical symptoms	nose	S	33A	SLV100
28448	mouse (C57BL/6) 2	2006	Germany	DKFZ	no clinical symptoms	nose	S	33A	SLV100
33645	mouse (C57BL/6) 3	2008	Germany	DKFZ	no clinical symptoms	nose	S	33F	SLV100
32276	mouse (C57BL/6) 4	2007	Germany	DKFZ	no clinical symptoms	nose	S	33A	SLV100
32969	mouse (C57BL/6) 5	2007	Germany	DKFZ	no clinical symptoms	nose	S	33A	SLV100
32970	mouse (C57BL/6) 6	2007	Germany	DKFZ	no clinical symptoms	nose	S	33A	SLV100
24072	rat (F344) 1	2003	Germany	DKFZ	no clinical symptoms	nose	S	14	SLV 124
24075	rat (F344) 2	2003	Germany	DKFZ	no clinical symptoms	nose	S	14	SLV 124
24077	rat (F344) 3	2003	Germany	DKFZ	no clinical symptoms	nose	S	14	SLV 124
24079	rat (F344) 4	2003	Germany	DKFZ	no clinical symptoms	nose	S	14	SLV 124
32965	rat 5	2007	Austria	VML	respiratory problems, sudden death	lung	S	19A	3546
34828	rat 6	2008	Monaco	VML	no data	wound	S	19A	SLV 3546
24070	guinea pig 1	1986	Germany	DKFZ	peritonitis, sepsis	lung	S	19F	new
24071	guinea pig 2	1986	Germany	DKFZ	peritonitis	abdomen	S	19F	new
28614	guinea pig 3	2006	The Netherlands	VML	severe respiratory problems	nose	S	19F	new
28976	guinea pig 4	2006	Germany	VML	head wound	wound	S	19F	new
31598	guinea pig 5	2007	Germany	VML	umbillical abcess, jaw abcess	abcess	S	19F	new
32066	guinea pig 6	2007	Germany	VML	respiratory problems, clotted nose	nose	S	19F	new
32449	guinea pig 7	2007	Germany	VML	purulent efflux nose, eyes	nose	S	19F	new
32668	guinea pig 8	2007	Germany	VML	coughing, nasal discharge, swollen lymphnodes	nose	S	19F	new
32669	guinea pig 9	2007	Germany	VML	coughing, nasal discharge	nose	S	19F	new
33098	guinea pig 10	2007	France	VML	tired, tilted head	ear	S	19F	new
33099	guinea pig 11	2007	Germany	VML	otitis media	ear	S	19F	new
34603	guinea pig 12	2008	The Netherlands	VML	nasal discharge, tiredness	nose	S	19F	new
34719	guinea pig 13	2008	Germany	VML	sneezing, nasal discharge	nose	S	19F	new
34728	guinea pig 14	2008	Germany	VML	Abortion of offspring, dead	Trachea, uterus, liver	S	19F	new
35456	guinea pig 15	2008	Peru	VML	no data	nose	S	19F	new
34950	guinea pig 16	2008	Germany	VML	recidiving rhinitis	nose	S	19F	new
35100	guinea pig 17	2008	Germany	VML	coughing, purulent nasal discharge	nose	S	19F	new
35489	guinea pig 18	2006	The Netherlands	VMDC	no data	no data	S	19F	new
35494	guinea pig 19	2007	The Netherlands	VMDC	respiratory problems	bulla tympanica	S	19F	new
28615	cat 1	2006	The Netherlands	VML	severe respiratory problems	nose	S	3	180
31057	cat 2	2007	Germany	VML	otitis media	wound	S	19F	177
32317	cat 3	2007	Germany	VML	conjunctivitis, strong sniffing	throat	S	6A	473
32667	cat 4	2007	Germany	VML	rhinitis, conjunctivitis	nose	S	19F	1815
32827	cat 5	2007	Germany	VML	dyspnoea, lungoedema,	thorax puncture	S	22F	819
33211	cat 6	2008	Germany	VML	purulent otitis externa	ear	S	14	9
34829	cat 7	2008	Deutschland	VML	nasal efflux	nose	S	31	1994
35135	cat 8	2008	The Netherlands	VML	sneezing, nasal discharge	nose	S	19A	3017
35488	cat 9	2003	The Netherlands	VMDC	purulent meningitis, died	brain	S	23F	311
35490	cat 10	2003	The Netherlands	VMDC	chronic rhinitis	nose	S	6B	176
35492	cat 11	1997	The Netherlands	VMDC	polyarthritis	synovia	S	22A	3705
35493	cat 12	2004	The Netherlands	VMDC	liquothorax, respiratory problems	thorax fluid	S	19F	177
32966	dog 1	2007	Netherlands	VML	coughing, sneezing, nasal discharge	nose	S	14	124
33437	dog 2	2008	Germany	VML	chronic recidivating rhinitis	nose	S	3	180
33770	dog 3	2008	Germany	VML	sinusitis	nose	S	10A	SLV1551
29800	horse 1	2006	Germany	VML	sinusitis	nose	S	3	new
32450	horse 2 (pony)	2007	Germany	VML	coughing, nasal discharge	nose	S	3	new
34368	horse 3	2008	Germany	VML	no data	nose, tracheallavage	S	3	new
34604	horse 4 (racing horse)	2008	Germany	VML	coughing, nasal discharge, swollen lymphnodes	BAL	S	3	new
24186	dolphin 1	1986	Germany	ZN	died withour disease symptoms	biopsy lung	S	23F	440
24187	dolphin 2	1986	Germany	ZN	died withour disease symptoms	biopsy lung	S	23F	440
35491	ape (gorilla)	2008	The Netherlands	ZK	acute infection, inflammated tonsilli with pus excretion	pus	S	14	124

a DKFZ: German Cancer Research Center (DKFZ), Heidelberg, Germany; VML: Vet Med Labor GmbH, Ludwigsburg, Germany, VMDC: Department of Infectious Diseases and Immunology (VMDC) of the Faculty of Veterinary Medicine of the University of Utrecht, the Netherlands, ZN: Zoo Nuremberg, Germany, ZK: Zoo Kerkrade, The Netherlands.

Further characterization of the streptococcal isolates at the German National Reference Center for Streptococci confirmed identification of all isolates as *Streptococcus pneumoniae*. All isolates were bile soluble, showed hybridization with a species-specific gene probe and had positive pneumolysin PCRs. Eighteen isolates, all from mastomys, were optochin-resistant.

All mastomys isolates (n = 32) were serotype 14. Guinea pig isolates were 19F, mice isolates were 33A or 33F, laboratory rat isolates were14, pet rat isolates were 19A, horse isolates were 3, dolphin isolates were 23F and the gorilla isolate was 14. Cats and dogs had a large variety of different serotypes (3 (n = 2), 6A, 6B, 10A, 14 (n = 2), 19A, 19F (n = 3), 22A, 22F, 23F, 31) (**Table 1**).

To exclude a possible transfer of *S. pneumoniae* from the animal facility staff members to the laboratory animals, throat swabs of animal facility staff members of the German Cancer Research Center (DKFZ) were taken. None of the seven staff members were found to carry pneumococci. All obtained isolates belonged to normal throat flora (*Streptococcus oralis*, *Streptococcus mitis*, *Streptococcus parasanguis* and *Gemella morbillorum*). No indications for the presence of pathogenic species were found.

To determine whether mastomys could carry pneumococcal isolates asymptomatically, four animals were followed over periods of several weeks. *S. pneumoniae* could be isolated from the same obviously healthy animal over periods of 6, 7, 17 and 22 weeks respectively (**Table 2**). All isolates were serotype 14.

**Table 2:** Carriage of *S. pneumoniae* by mastomys.

Isolate number	Animal	1st sample (week of life)	last sample (week of life)	Carriage length (weeks)	Year of isolation	country	origin	Diagnosis	Site of isolation	Opto Test	Serotype	MLST
31578	mastomys #18	52	74	22	2007	Germany	DKFZ	no clinical symptoms	eye	S	14	n.d.
32161	mastomys #29	66	73	7	2007	Germany	DKFZ	no clinical symptoms	eye	S	14	n.d.
31467	mastomys #33	55	72	17	2007	Germany	DKFZ	no clinical symptoms	eye	S	14	n.d.
31580	mastomys #168	45	51	6	2007	Germany	DKFZ	no clinical symptoms	eye	S	14	n.d.

a DKFZ: German Cancer Research Center (DKFZ), Heidelberg, Germany; n.d.: not done.

MIC analysis of the isolates showed that four isolates were resistant to clarithromycin, three of them were also resistant to clindamycin and tetracyclin. The isolates were from two cats and two pet rats. The three multiresistant isolates showed an iMLS_B_ phenotype and an *ermB* genotype, the single resistant isolate had the M-phenotype, and *mef(A)* subclass *mef(A)* (**Table 3**). All other isolates were susceptible for all tested substances (penicillin G, amoxicillin, cefotaxime, cefpodoxime, cefuroxime, clarithromycin, clindamycin, tetracycline, levofloxacin, trimethoprim-sulfamethoxazole and vancomycin).

**Table 3:** Macrolide resistant isolates found in pet cats and rats.

Animal	Diagnosis	MLST	Serotype	Macrolide resistance genotype	Macrolide resistance phenotype	CLA (μg/ml)	CLI (μg/ml)	TET (μg/ml)	PEN (μg/ml)	CEF (μg/ml)
cat 4	rhinitis, conjunctivitis	1815	19F	*ermB*	iMLS_B_	16	16	16	0,015	0,015
cat 6	otitis externa eitrig	9	14	*mefA*	M	8	0,12	0,25	0,015	0,015
rat 5	Breathing problems, sudden death	3546	19A	*ermB*	iMLS_B_	16	8	16	0,015	0,015
rat 6	no data	SLV 3546	19A	*ermB*	iMLS_B_	16	16	16	0,06	0,015

CLA: clarithromycin, CLI: clindamycin, TET: tetracyclin, PEN: penicillin, CEF: cefotaxime.

MLST analysis revealed that all mastomys isolates were sequence type (ST)15. All 19 guinea pigs had STs with the same new combination of existing alleles, which has not been found previously, nor have single or double locus variants (**Table 4**). Mice isolates showed two new sequence types, both single locus variants (SLV) of ST100, one harbouring a variant *spi* allele, with 99% identity to allele 9, and the other a variant *recP* allele (99% identity to allele 12). The four laboratory rats had the same SLV of ST124, showing a variant *gki* allele (99% identity to allele 1). The cats and dogs showed a large variety of STs (9, 124, 176, 177 (n = 2), 180 (n = 2), 311, 473, 819, 1815, 1994, 3017, 3705 and an SLV of 1551). Three horses had the same new ST, with new alleles for *spi* (99% identity to allele 10), *xpt* (99% identity to allele 1) and *ddl* (99% identity to allele 6). The fourth horse (a pony horse) had the same ST, except for the *spi* allele which was an exact match with allele 10. Both dolphin isolates had ST 440, the gorilla isolate ST 124 (**Table 4**).

**Table 4:** MLSTs of 81 *Streptococcus pneumoniae* isolates from pets and laboratory animals in Germany.

Animal	number of animals	Year	Serotype	MLST	*aroE*	*gdh*	*gki*	*recP*	*spi*	*xpt*	*ddl*
mastomys	28	2003–2006	14	15	1	5	4	5	5	3	8
guinea pig	19	1986/2006/2007/2008	19F	new	2	5	4	5	27	20	5
mouse (C57BL/6)	5	2006	33A/33F	new (SLV 100)	5	12	29	12	9 (99%)	39	18
mouse (C57BL/6)	1	2006	33A	new (SLV 100)	5	12	29	12 (99%)	9	39	18
rat (F344)	4	2003	14	new (SLV 124)	7	5	1 (99%)	8	14	11	14
rat (pet)	1	2007	19A	3546	1	5	41	5	10	28	8
rat (pet)	1	2008	19A	new (SLV 3546)	1	5	41	5	10	99% 28	8
cat	1	2008	14	9	1	5	4	5	5	1	8
cat	1	2003	6B	176	7	13	8	6	10	6	14
cat	2	2004/2007	19F	177	7	14	4	12	1	1	14
cat	1	2006	3	180	7	15	2	10	6	1	22
cat	1	2003	23F	311	1	8	9	1	6	4	6
cat	1	2007	6A	473	7	25	4	4	15	20	28
cat	1	2007	22F	819	1	1	4	1	18	58	18
cat	1	2007	19F	1815	1	5	4	12	5	3	159
cat	1	2008	31	1994	1	2	29	1	111	14	18
cat	1	2008	19A	3017	8	11	14	1	17	230	14
cat	1	1997	22A	3705	2	194	53	18	10	1	1
dog	1	2007	14	124	7	5	1	8	14	11	14
dog	1	2008	3	180	7	15	2	10	6	1	22
dog	1	2008	10A	SLV 1551	5	7	4	6	10	1	6
horse	3	2006	3	new	10	9	4	12	10 (99%)	10 (99%)	6 (99%)
horse (pony)	1	2007	3	new	10	9	4	12	10	10 (99%)	6 (99%)
dolphin	2	1986	23F	440	7	5	1	1	13	31	14
gorilla	1	2008	14	124	7	5	1	8	14	11	14

ST 15/serotype 14, ST 100/33F, ST 124/14, ST 3546/19A and ST 440/23F found in mastomys, mice, rats, dog, gorilla and dolphins respectively are common sequence type/serotype combinations found in human pneumococcal isolates (www.mlst.net). This is also the case for the combinations ST 9/14, ST 176/6B, ST 177/19F, ST 180/3, ST 311/23F, ST 473/6A, ST 819/22F, ST 1551/10A, ST 1815/19F, ST 1994/31, and ST 3017/19A found in cats and dogs. The combination ST 3705/22A found in one cat has so far never been encountered in humans.

## Discussion

It is generally accepted that *Streptococcus pneumoniae* is strictly a human pathogen despite there being animal models (mouse, rat) available to study pneumococcal infections. However, on the other hand, recent literature describing naturally occurring carriage of or infections by pneumococci in animal hosts is scarce [13], [14]. A report from 1988 describes the isolation of *S. pneumoniae* serotype 3 from a racehorse [15]. Other pneumococcal isolates from racehorses also appear to have serotype 3 [16], [17]. In a recent report from Chi *et al. S. pneumoniae* isolated from Chimpansees are described [13]. Notably, anecdotal reports of *S. pneumoniae* isolated from animals can be found as early as in the 1940s (**Table 5**).

**Table 5:** Anecdotal reports of *Streptococcus pneumoniae* isolated from different mammalian species.

Animals	Number of infected animals	Outbreak	Serotypes	Laboratory animals	Year	Source
guinea pigs	39	yes	19	yes	1945	Homburger *et al.* [33]
rats	71	yes	2	yes	1950	Mirick *et al*. [18]
rats	254	yes	8	yes	1965	Ford [19]
rats	156	no	2, 3, 19	yes	1969	Weisbroth and Freimer [20]
rats	32	no	3	yes	1971	Mitruka [21]
mice, rats	9,10	yes	35	yes	1988	Fallon et al. [34]
horses	10	no	3	no	1988	Huber and Willoughby [15]
horses	11	no	3	no	1999	Whatmore et al. [16]
cat	1	no	n.d.	no	2006	Zhang et al. [14]

n.d: no data.

Outbreaks of infections with *S. pneumoniae* of serotypes 2, 3, 8 and 19 in laboratory rats in the US have been described in several publications [18], [19], [20], [21]. Furthermore, veterinary textbooks describe pneumococcal infections to be not only present in rodents (rats, mice, guinea pigs) but also in larger domestic animals (calves, horses) [22], [23].

Since all pneumococcal isolates obtained from animals in this study were bile soluble, and showed hybridization with a species specific DNA probe, they were unambiguously identified as *S. pneumoniae*. Moreover, the fact that serotyping and MLS typing gave positive results confirms that the isolates are pneumococci. However, 18 out of 32 mastomys isolates were optochin-resistant. Since pneumococcal isolates are almost always optochin susceptible and optochin resistance is usually a trait of other viridance group streptococci, this could be one of the reasons why pneumococci are being overlooked and therefore not reported in animal isolates, thereby leading to underrepresentation of information on the extent of pneumococci colonization in the literature. Finally, it is noteworthy that optochin-resistant pneumococci are being increasingly reported from human sources [24].

The most obvious cause of infection for the laboratory animals would be the animal facility staff. For the mastomys colony in the German Cancer Research Center this could be verified. None of the staff carried pneumococci at the moment of sampling, making it less likely that animals had been infected recently. However, it cannot be excluded that the colony got infected at an earlier point in time since carriage in humans is transient. For the other laboratory animals, which came from large commercial facilities, a possible infection by facility staff members could not be tested.

Most ST/serotype combinations identified in the isolates in this study are commonly found among human pneumococcal isolates (www.mlst.net). The combination ST 15/serotype 14, found in 28 mastomys is commonly found among human isolates in association with meningitis and bacteremia and has been reported from the UK, the Netherlands, Portugal, Germany, Italy, Brazil and Hong Kong (www.mlst.net).

The combination ST 100/33F is commonly encountered among human isolates and has been reported from Spain, Germany, Poland and USA. The combination with serotype 33A has not been reported so far (www.mlst.net). This could indicate that a serotype switch from 33F to 33A has occurred in mice, as well as a mutation in the *spi* or the *recP* allele.

The combination ST 124/serotype 14 found in the gorilla, a dog and, as an SLV, in the laboratory rats has been reported from Germany, Sweden, UK, Denmark, Finland, Norway, The Netherlands, Australia, Canada, and Poland and is associated with meningitis, bacteremia, pneumonia and otitis media in human isolates, as well as with carriage (www.mlst.net).

The combination ST 3546/19A found in two pet rats has been reported only once, from Norway, and was isolated from blood (www.mlst.net).

The combination serotype ST 440/23F (UK, Poland, Czech Republic, Switzerland) found in the two dolphins is also commonly encountered among human isolates where it is associated with meningitis and bacteremia as well as being found in carriage (www.mlst.net).

The combination ST 180/serotype 3 found in a cat and in a dog isolate has been reported from several countries (UK, Spain, Poland, Portugal, Italy, Taiwan, Canada, Denmark, Netherlands) and is associated with invasive disease (meningitis, bacteremia) but also with community acquired pneumonia, otitis media and carriage (www.mlst.net). The combinations ST 9/14, ST 176/6B, ST 177/19F, ST 311/23F, ST 473/6A, ST 819/22F, ST 1551/10A, and ST 1994/31 found in cats and dogs are all well known human ST/serotype combinations associated with both invasive disease and carriage (www.mlst.net). Two isolates, ST 1815/19F and ST 3017/19A, both found in cats have been reported only once to the MLST database (www.mlst.net). ST 1815/19F was from a case of bacteremia from the Czech Republic. ST 3017/19A was isolated from blood from a patient from the Netherlands, which is also the country of origin of the cat. The combination ST 3705/22A has not been reported to the MLST database. The only isolate with ST 3705 is a carriage isolate from the Netherlands with serogroup 19. Again this isolate is from the same country as the cat in this study.

eBURST analyses revealed that STs 15, 124, 177, 180, 473 and 1994 are all predicted founders of clonal complexes (CC). ST 124 is the predicted founder of a group of 78 STs and is one of the original penicillin-resistant clones described in South Africa [25]. CC124 also comprises ST 440. ST9 and ST1815 belong to CC15. STs 176, 311, 819, 1551, and 3705 are members of different clonal complexes. STs 3546 and 3017 are singletons, i.e. they are not related to any other STs in the database. Taken together, our data show that the pneumococcal isolates from mastomys, mice, rats, cats, dogs, dolphins and gorilla analysed in this study are highly similar, if not identical, to human isolates related to invasive pneumococcal disease and carriage.

Serotype 19F, found in all four guinea pig isolates, is a common serotype found in human isolates (www.mlst.net), where it is associated with invasive pneumococcal disease as well as carriage. It occurs in combination with large numbers of different STs. However, the STs of all 19 guinea pig isolates consist of a new allele combination never encountered in human isolates. From these findings it may be interpreted that the guinea pigs did not pick up the pneumococcal infections from humans. Moreover, the fact that the same new allele combination has been found in isolates from guinea pigs from different locations (pets, laboratory animals), different countries (France, Germany, Peru, the Netherlands) and widely separated in time (1986, 2006) seems to indicate that this clone is typical for guinea pigs and that these animals are a reservoir for *S. pneumoniae* of this specific sequence type. As apparent from our diagnostic data, this clone is clearly pathogenic for guinea pigs.

The four horse isolates described here have serotype 3 and a new ST, with three or two new alleles. Whatmore *et al.* report on a collection of horse pneumococcal isolates of serotype 3 and conclude on the basis of RFLP analyses that these isolates are closely related to, but distinguishable from human pneumococcal isolates [16]. Showing a new sequence type, our data confirm that the horse isolates form a population different from human isolates. Pneumococci found in guinea pigs and horses seem to belong to separate host specific populations. The isolates found in mastomys, mice, rats, cats, dogs, dolphins and gorilla are most likely identical to human pneumococcal isolates.

Four animals, two cats and two rats, had macrolide resistant isolates. One cat isolate showed moderate resistance to clarithromycin and carried the *mefA* subclass *mef(A)* gene. The isolate had ST15 combined with serotype 14, which is the most common macrolide resistant clone (PMEN England^14^-9) in Germany [26], [27]. The other isolates all contained the *ermB* resistance marker, which results in higher MICs for clarithromycin and resistance to clindamycin. These isolates were also resistant to tetracyclin. The ST/serotype combinations were 1815/19F and (SLV)3546/19A. As discussed above these are rather rare combinations. However, serotypes 19A and 19F are strongly associated with multidrug resistance.

Our findings are unequivocal proof that pneumococci are capable of causing severe disease in a number of different animal species. One of the pet guinea pigs was a member of a larger colony in which the animals suffered from respiratory problems and of which several animals died. Further, the cats, dogs and horses also suffered from severe respiratory problems, and the two dolphins were likely to have died from pneumococcal infections.

On the other hand, laboratory animals (mastomys, mice and rats) from which pneumococci were isolated showed no disease symptoms. For two animals from the mastomys colony that suffered from conjunctivitis, another potential causative agent for the clinical signs (*Pasteurella pneumotropica* biotype Heyl) was found in large numbers in addition to *S. pneumoniae*. This finding indicates that the rodents may carry *S. pneumoniae* in their normal flora without suffering from pneumococcal disease, much like in the case of humans. Indeed, for four individual mastomys, we were able to isolate the same isolate over a period of up to 22 weeks, showing that the animals are carriers.

This is the first time that a larger collection of *S. pneumoniae* isolated from a range of animals has been characterized on a molecular basis using MLST, enabling a solid comparison to human isolates present in the MLST database. The results show that on one hand animals can be infected with human pneumococcal clones. On the other hand animals like horses and guinea pigs seem to be affected by their ‘own’ specific pneumococcal clones. The fact that pets like cats, dogs, guinea pigs, rats and mice are possible carriers of pneumococcal strains, and in many cases of the same ST/serotype combinations as human isolates, is worrisome. Even more alarming is the fact that antibiotic resistant strains, on one occasion even the most common macrolide resistant clone in Germany (England^14^-9), could be isolated from pets. Our data imply that there is an infection route for pneumococci from humans to pet animals that live in close contact to humans, especially children. By residing in animals, pneumococci would not only enlarge their gene-pool with other streptococcal species found more commonly in animals, but would also be able to avoid the negative selective pressure of vaccination in children. The question remains if pneumococci residing in animals can re-infect humans. On basis of the present data no conclusion on this can be drawn and further research is needed.

## Materials and Methods

### Study material

The German National Reference Center for Streptococci (GNRCS) received a total of 85 bacterial isolates from animals in Austria, France, Germany, Monaco, The Netherlands and Peru in 1986 (n = 4) and during 2003–2008 (n = 81). Fourty-four isolates were from the animal facility of the German Cancer Research Center (DKFZ) in Heidelberg, Germany, 32 isolates were from pets treated at a commercial veterinary medical laboratory (Vet Med Labor GmbH) in Ludwigsburg, Germany, 6 isolates were obtained from the Department of Infectious Diseases and Immunology (VMDC) of the Faculty of Veterinary Medicine of the University of Utrecht, the Netherlands, two isolates were from a zoo in Nuremberg and one from a zoo in Kerkrade, the Netherlands. All samples were sent to the GNRCS as isolated bacteria, no primary material was received.

### Testing of animal facility staff

Throat swaps were obtained in May 2006 from seven members of the animal facility staff of the German Cancer Research Center and tested for bacterial content using routine microbiological methods. Throat swabs were taken by the institute medical officer of the DKFZ, after verbal consent of each of the animal facility staff members. Isolated bacteria were analysed both at the DKFZ as well as at the GNRCS.

### Characterization of isolates

*Streptococcus pneumoniae* isolates were characterized by optochin susceptibility and bile solubility testing. Optochin susceptibility testing was carried out in a 5% CO_2_ atmosphere on sheep blood agar [28]. Bile solubility testing was performed by preparing a bacterial suspension in 1 mL 0.85% NaCl (McFarland standard 1.0) adding four drops of 10% sodium deoxycholate. Complete lysis after incubation for 2 h at 35°C was taken as a positive result. A commercially available DNA hybridization test (AccuProbe® *Streptococcus pneumoniae* identification test; bioMérieux, Germany) was used according to the manufacturer's instructions.

### Serotyping

Pneumococcal strains were serotyped by the Neufeld Quellung reaction using type and factor sera provided by the Statens Serum Institut, Copenhagen, Denmark [29].

### Susceptibility testing

Minimal inhibitory concentration (MIC) testing was performed using the broth microdilution method as recommended by the Clinical Laboratory Standards Institute [30]. Microtiter plates containing penicillin G, amoxicillin, cefotaxime, cefpodoxime, cefuroxime, clarithromycin, clindamycin, tetracycline, levofloxacin, trimethoprim-sulfamethoxazole and vancomycin with cation-adjusted Mueller-Hinton broth (Oxoid, Wesel, Germany) plus 5% lysed horse blood (Oxoid) were used. The final inocculum was 5×10^5^ CFU/ml. MICs were determined following incubation for 24 h at 35°C in ambient air. *S. pneumoniae* ATCC 49619 was used as a control strain. Current Clinical Laboratory Standards Institute interpretive criteria were used to define antimicrobial resistance [30].

### DNA extraction

Isolates were inoculated from agar plates (5% sheep blood) into a sterile culture tube containing 10 mL Todd-Hewitt broth (Oxoid Limited Basingstoke, Hampshire, UK) and incubated over night at 37°C. After centrifugation, the chromosomal DNA was isolated using a DNA extraction kit (Qiagen, Hilden, Germany).

### Pneumolysin PCR

Analysis of the pneumolysin gene (*ply)* was performed using real-time PCR on a *Light Cycler* (Roche Diagnostics GmbH, Penzberg, Germany) according to the LightCycler Operator's Manual Version 3.5. PCR was carried out using the *Light Cycler Faststart DNA Master SYBR Green I* – kit according to the manufacturer's instructions. The reaction mixture of 20 µl contained 2 µl SYBR Green I, 2 µl MgCl_2_, 12 µl H_2_0, 1.5 µl of each primer and 200 ng of DNA. The oligonucleotide primers pair was ply ^fwd 894–915^ 5′ TGC AGA GCG TCC TTT GGT CTA T 3′ and ply ^rev 974–950^ 5′ CTC TTA CTC GTG GTT TCC AAC TTG A 3′ [31]. PCR cycling comprised initial denaturation for 10 min at 95°C and 35 amplification cycles for 0 s at 95°C, 2 s at 62°C and 4 s at 72°C with temperature transition rates of 20°C/s. This was followed by a melting programme of 30 s at 95°C, 30 s at 67°C and 0 s at 95°C at rates of 20, 20, and 0.1°C/s, respectively, and cooling at 40°C for at least 30 s.

### Multilocus sequence typing

Multilocus sequence typing (MLST) of 81 isolates was carried out as described previously. Briefly, internal fragments of the *aroE*, *gdh*, *gki*, *recP*, *spi*, *xpt* and *ddl* genes were amplified by PCR from chromosomal DNA with the primer pairs described by Enright and Spratt [12]. The alleles at each of the seven loci provide the allelic profile of each isolate and also define their sequence type (ST). Allelic profiles are shown as the alleles at each of the seven loci, in the order *aroE*, *gdh*, *gki*, *recP*, *spi*, *xpt* and *ddl*.

### MLST analysis

STs were compared to the pneumococcal MLST database on www.mlst.net. The STs were analysed using the program eBURST. This program is able to predict and display the relationships between closely-related isolates of a bacterial species or population. eBURST, unlike cluster diagrams, trees or dendrograms, uses a simple but appropriate model of bacterial evolution in which an ancestral (or founding) genotype increases in frequency in the population, and while doing so, begins to diversify to produce a cluster of closely-related genotypes that are all descended from the founding genotype. This cluster of related genotypes is referred to as a ‘clonal complex’ (eburst.mlst.net) [32].
